# The *NPM1* Mutation Type Has No Impact on Survival in Cytogenetically Normal AML

**DOI:** 10.1371/journal.pone.0109759

**Published:** 2014-10-09

**Authors:** Friederike Pastore, Philipp A. Greif, Stephanie Schneider, Bianka Ksienzyk, Gudrun Mellert, Evelyn Zellmeier, Jan Braess, Cristina M. Sauerland, Achim Heinecke, Utz Krug, Wolfgang E. Berdel, Thomas Buechner, Bernhard Woermann, Wolfgang Hiddemann, Karsten Spiekermann

**Affiliations:** 1 Laboratory for Leukemia Diagnostics, Dept. of Internal Medicine III, University Hospital Munich Großhadern, Ludwig-Maximilian-University (LMU), Munich, Germany; 2 Clinical Cooperative Group Pathogenesis of Acute Myeloid Leukemia, Helmholtz Zentrum München, German Research Center for Environmental Health, Munich, Germany; 3 German Cancer Consortium (DKTK), Heidelberg, Germany; 4 German Cancer Research Center (DKFZ), Heidelberg, Germany; 5 Dept. of Oncology and Hematology, Krankenhaus Barmherzige Brüder, Regensburg, Germany; 6 Institute of Biostatistics and Clinical Research, University of Muenster, Muenster, Germany; 7 Dept. of Internal Medicine A, Hematology and Oncology, University of Muenster, Muenster, Germany; 8 German Society of Hematology and Oncology, Berlin, Germany; University of Texas MD Anderson Cancer Center, United States of America

## Abstract

*NPM1* mutations represent frequent genetic alterations in patients with acute myeloid leukemia (AML) associated with a favorable prognosis. Different types of *NPM1* mutations have been described. The purpose of our study was to evaluate the relevance of different *NPM1* mutation types with regard to clinical outcome. Our analyses were based on 349 *NPM1*-mutated AML patients treated in the AMLCG99 trial. Complete remission rates, overall survival and relapse-free survival were not significantly different between patients with *NPM1* type A or rare type mutations. The *NPM1* mutation type does not seem to play a role in risk stratification of cytogenetically normal AML.

## Introduction

The gene encoding nucleophosmin (*NPM1*), a nucleo-cytoplasmatic shuttling protein with prominent nucleolar localization is located on 5q35.1 and contains 12 exons. *NPM1* is involved in epigenetic control (binding of nucleic acids, centrosome duplication), ribosomal protein assembly as molecular chaperon and regulation of the ARF-p53-tumor suppressor pathway [1], [2].

*NPM1* mutations can be found in 35% of patients with cytogenetically normal acute myeloid leukemia (AML). They are associated with a favorable prognosis in the absence of an additional internal tandem duplication in the fms-related tyrosine 3 gene (*FLT3-ITD*), female gender, high white blood count (WBC), high amount of bone marrow blasts as well as a high platelet count [1].

In the vast majority of cases *NPM1* mutations result in a frameshift due to an insertion of four bases, which cluster in exon 12. Different types of *NPM1* mutations have been described according to the inserted tetranucleotide, the most common being type A mutations (TCTG) in 80%, followed by type B (CATG) and type D (CCTG) mutations in about 10%, and a spectrum of other mutations accounting for 10% of cases. In rare cases, insertions from 2 to 9 bases can occur (e.g. types E, F) [1].

The altered *NPM1* protein (*NPM1*c+) contains an additional C terminal nuclear export signal (NES) motif and loses at least one tryptophan residue, causing an aberrant cytoplasmatic localization of the protein [3].

Little is known about the prognostic relevance of the variable *NPM1* mutation types. In *FLT3-ITD* negative patients, a Korean study found an adverse impact on overall survival (OS) and duration of complete remission (CR) in *NPM1* mutations other than type A [4]. In *FLT3-ITD* positive patients, another recent study reported an adverse OS in *NPM1* type A mutations [5] whereas - in contrast to Koh et al. - they did not observe an effect of *NPM1* mutation type in *FLT3-ITD* negative patients.

We aimed at studying the clinical relevance of *NPM1* mutation types on prognosis in a large homogeneously treated cohort of cytogenetically normal AML patients.

## Patients and Methods

### Patients

Our analyses were based on a total of 783 patients with newly diagnosed cytogenetically normal AML treated within the randomized multicenter German AML Cooperative Group 99 (AMLCG99) trial comparing a double induction therapy with thioguanine, cytarabine and daunorubicin (TAD) and high dose mitoxantrone (HAM) versus HAM-HAM [6].

The AMLCG99 trial is registered at ClinicalTrials.gov (NCT00266136) and was approved by the local institutional review boards of all participating centers. Informed consent was obtained from all patients in accordance with the Declaration of Helsinki.

### Cytogenetic and molecular analyses

All cytogenetic and molecular analyses were performed on bone marrow aspirates. Cytogenetic analyses were based on the assessment of at least 20 metaphases and performed according to the international system of cytogenetic nomenclature (ISCN) guidelines [7]. Mutations of *NPM1* [1], *FLT3-ITD* [8], the *FLT3-ITD* mRNA level [9], [10], *CEBPA* [11], [12], were analyzed as previously described. The nomenclature of the *NPM1* mutation type was performed according to the literature [1], [13], [14]. *NPM1* mutations other than type A mutations were grouped together as rare type mutations (*NPM1*-rare), *NPM1* mutations other than type A, B or D were grouped together as other mutations (*NPM1*-other).

### Outcome Parameters

OS was calculated from the date of randomization until death from any cause. Surviving patients were censored at the date of last follow-up. Relapse-free survival (RFS) was determined for responders from the first day of CR until relapse or death from any cause. Patients in CR that did not experience a relapse were censored at the last disease assessment.

For patients who had undergone allogeneic transplantation according to the study protocol, OS and RFS times were censored at the time of transplantation.

### Statistical Analysis

Continuous characteristics between patient groups were compared using the Mann-Whitney U test for 2 groups and the Kruskal-Wallis test for 3 groups. The 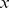 ^2^-test for was applied for categorical variables. Kaplan-Meier estimation for OS and RFS was performed comparing *NPM1*-mutated patients with a type A mutation (*NPM1-A*) versus *NPM1*-mutated patients with another type of *NPM1* mutation (rare type: *NPM1-RA*) as well as both groups combined with either *FLT3-ITD* or without *FLT3-ITD*. In a second analysis, patients with *NPM1* mutations types A, B and D were analyzed independently and compared to patients carrying other mutations than A, B and D who were grouped together as “*NPM1*- other”.

Hazard ratios and 95% confidence intervals between the different groups were estimated by Cox regression analyses.

Independent prognostic parameters for OS and RFS were identified by univariable Cox regression analyses and a multivariable Cox regression model using backward elimination with an exclusion significance level of 5%. For all analyses, a p-value<0.05 was considered significant. For the estimation of the median follow-up time, the reversed Kaplan-Meier method was applied [15].

## Results

### Patient characteristics

Analyses were performed in 349 *NPM1*-mutated patients of 667 patients with CN-AML treated within the AMLCG99 trial, after exclusion of 106 patients without information of the *NPM1* mutation status. For an overview of patient selection, please refer to **Figure S1 in File S1**.

Out of 349 patients, 16% underwent allogeneic stem cell transplantation in first CR. Median follow-up for OS was 45.3 months. Median OS was 45.6 months with 151 events. In 74% of patients who achieved a CR, median RFS was 37.1 months with 115 events.

Patient characteristics are summarized in **Table S1 in File S1**. The selected 677 patients from whom the 349 *NPM1*-mutated patients were analyzed displayed similar patient characteristics and outcome (OS and RFS, not significantly different) as the unselected patients.

The majority of patients (77%) carried *NPM1* type A mutations, followed by 9% with *NPM1* type D, 5% with *NPM1* type B mutations and 2% with *NPM1* type I mutations. All other mutations occurred in ≤1% of patients (**Table S2 in File S1**).

### *NPM1* mutation type does not influence outcome

#### A) *NPM1* mutation type A versus rare type mutations

**Figure 1:**
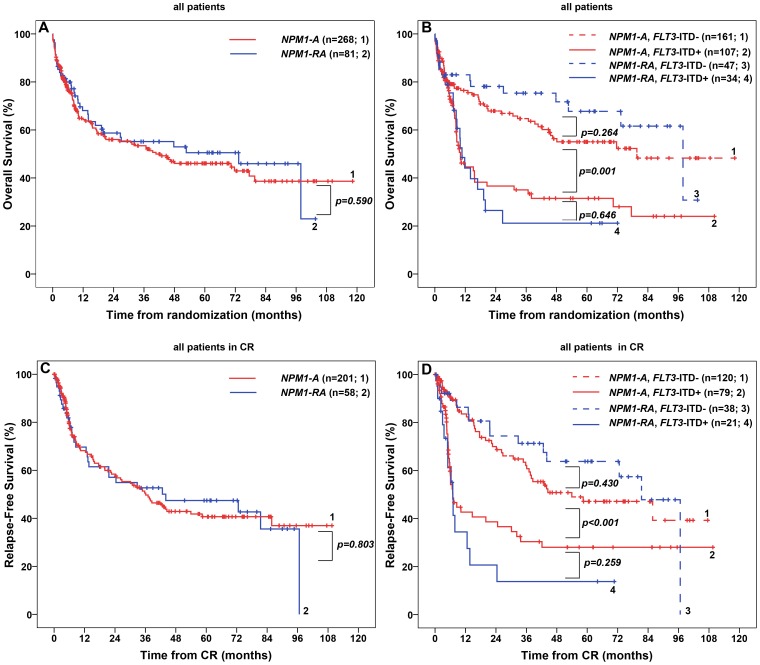
Overall Survival (OS) and Relapse-Free Survival (RFS) in 349 patients with cytogenetically normal acute myeloid leukemia and *NPM1* mutation treated in the AMLCG99 study. (A) OS in patients with *NPM1* type A mutation versus *NPM1* rare type mutation. (B) OS in patients with *NPM1* type A mutation versus *NPM1* rare type mutation with or without an additional *FLT3-ITD*. (C) RFS in patients with *NPM1* type A mutation versus *NPM1* rare type mutation. (D) RFS in patients with *NPM1* type A mutation versus *NPM1* rare type mutation with or without an additional *FLT3-ITD*. **Abbreviations**: CR, complete remission; *FLT3-ITD*+, presence of an internal tandem duplication in the fms-related tyrosine 3 gene; *FLT3-ITD*-, absence of an internal tandem duplication in the fms-related tyrosine 3 gene; *NPM1-A*, mutation in the nucleophosmin gene consisting of an insertion of the tetranucleotide TCTG; *NPM1-RA*, mutation in the nucleophosmin gene other than type A.

We did not observe any significant difference between patients with *NPM1 type* A and rare mutations in clinical or molecular parameters, in particular, there was no difference regarding OS and RFS (**Figures 1A and 1C** **, Table S1 in File S1**).

In patients with *NPM1* mutation (*NPM1+*), the presence of an *FLT3-ITD* (*FLT3-ITD*+) caused significant changes in WBC, LDH level, amount of bone marrow blasts and a decrease in OS and RFS compared to patients without *FLT3-ITD* (*FLT3-ITD*-) (**Figure S2 in File S1**).

We did not observe any difference in patient characteristics, OS or RFS between *NPM1-A*/*FLT3-ITD*- versus *NPM1-RA*/*FLT3-ITD*- patients (**Figure 1B and 1D** **, Table S1 in File S1**). Similar results were obtained for *NPM1-A*/*FLT3-ITD*+ versus *NPM1-RA*/*FLT3-ITD*+ patients, except for WBC which was higher in the rare type group (p = 0.005).

In multivariable Cox regression analysis, parameters with independent adverse impact on OS and RFS were older age, a high WBC and the presence of an *FLT3-ITD*. Importantly, the *NPM1* mutation type (*NPM1-A* versus *NPM1-RA*) was not significantly correlated with OS or RFS, neither in univariable, (**Table S3 in File S1**) nor in multivariable Cox regression models (**Table** **1**).

**Table 1:** Multivariable Cox regression models for OS and RFS in 349 *NPM1*-mutated patients.

independent prognostic factors	comparison	OS			RFS		
		HR	95% CI	p	HR	95% CI	p
Age	per 10 years	1.71	1.44–2.03	<0.001	1.55	1.28–1.87	<0.001
WBC	10^9^/L, per 10-fold increase	1.63	1.16–2.30	0.005	1.71	1.15–2.56	0.009
*FLT3-ITD*	positive versus negative	2.04	1.40–2.96	<0.001	2.45	1.60–3.75	<0.001
*NPM1* mutation type	*NPM1-A*	1.06	0.71–1.59	0.764	0.89	0.57–1.38	0.590
	versus						
	*NPM1-RA*						

White blood cell count, platelet count, hemoglobin level, lactase dehydrogenase level, bone marrow blasts, de novo AML versus non de novo AML, performance status, sex, age, type A versus rare type *NPM1* mutation, *FLT3-ITD*, monoallelic *CEBPA* mutations, biallelic *CEBPA* mutations were included in the Cox regression models for OS and RFS with backward elimination. The analyses were performed using 313 patients for OS and 227 RFS who had data for all these variables. A p-value of <0.05 was considered as indicating significant differences. All parameters that did not have a significant impact on OS or RFS are not shown in the table, except for the *NPM1* mutation type.

**Abbreviations**: *CEBPA*, CCAAT/enhancer-binding protein alpha gene; CI, confidence interval; *FLT3-ITD*, internal tandem duplication of the *FLT3* gene; HR, hazard ratio; negative, absence of *FLT3-ITD*; *NPM1*, nucleophosmin gene; *NPM1-A*, mutation in the nucleophosmin gene leading to the insertion of the tetranucleotide TCTG; *NPM1-RA*, mutation in the nucleophosmin gene other than type A; OS, Overall survival; p, p value; positive, presence of *FLT3-ITD*; RFS, Relapse-free survival; WBC, white blood cell count.

Similar results were obtained for the effect of *NPM1-A* versus *NPM1-RA* on OS and RFS in patients receiving chemotherapy only (without allogeneic transplantation in first CR), patients with de novo AML and patients <60 or ≥60 years (**text and Figures S3, S4 and S5 in File S1**).

#### B) *NPM1* mutation types A versus B versus D versus other mutations

**Figure 2:**
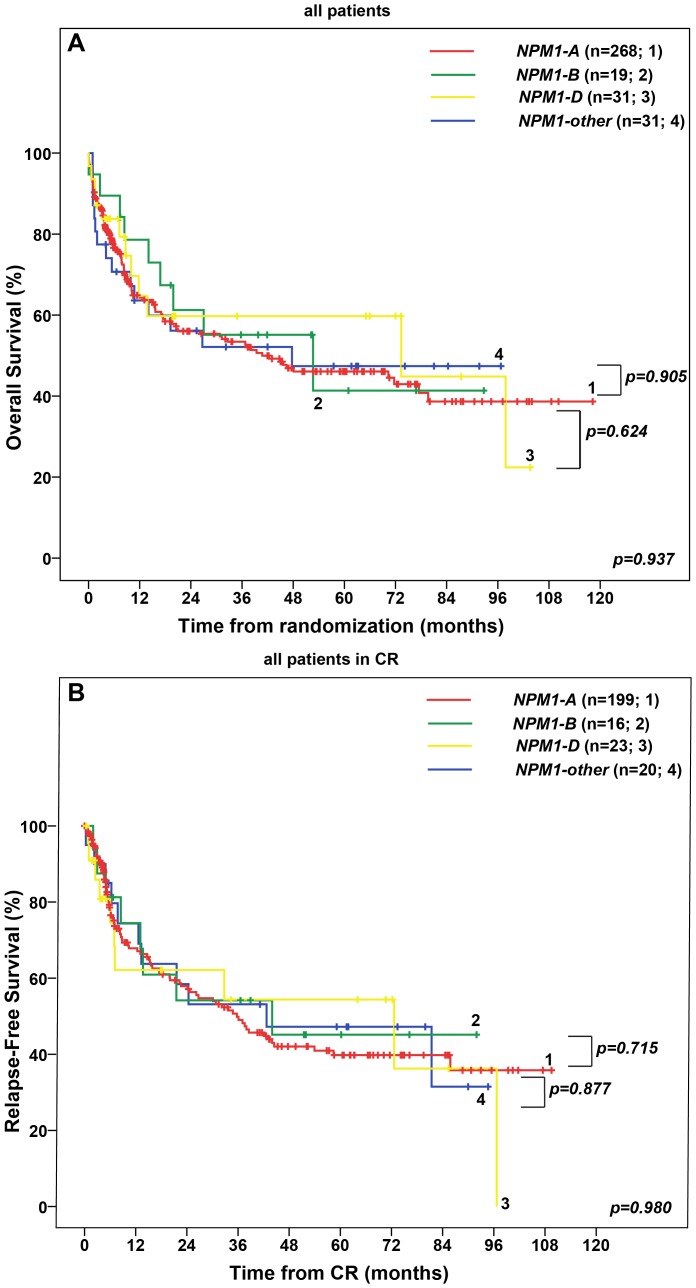
Overall Survival (OS) and Relapse-Free Survival (RFS) in 349 patients with cytogenetically normal acute myeloid leukemia and *NPM1* mutation treated in the AMLCG99 study. (A) OS in patients with *NPM1* type A mutation versus *NPM1* type B mutation versus *NPM1* type D mutation versus *NPM1* type other mutation. (B) RFS in patients with *NPM1* type A mutation versus *NPM1* type B mutation versus *NPM1* type D mutation versus *NPM1* type other mutation. **Abbreviations**: CR, complete remission; *FLT3-ITD*+, presence of an internal tandem duplication in the fms-related tyrosine 3 gene; *FLT3-ITD*-, absence of an internal tandem duplication in the fms-related tyrosine 3 gene; *NPM1-A*, mutation in the nucleophosmin gene consisting of an insertion of the tetranucleotide TCTG; *NPM1-B*, mutation in the nucleophosmin gene consisting of an insertion of the tetranucleotide CATG, *NPM1-D*, mutation in the nucleophosmin gene consisting of an insertion of the tetranucleotide CCTG, *NPM1-other*, mutation in the nucleophosmin gene other than *NPM1* mutation types A, B, D.

We did not find a statistical difference with regard to OS and RFS in patients with *NPM1* type A, B, D and other mutations (p = 0.937, p = 0.980) (**Figures 2A and 2B**). Furthermore, OS and RFS were similar between patients with type A, B, D and other mutations without an additional *FLT3-ITD* (data not shown). *NPM1-A/FLT3-ITD*+ patients showed similar OS and RFS as patients with *NPM1-B/FLT3-ITD*+, *NPM1-D/FLT3-ITD*+ and *NPM1-other/FLT3-ITD*+ patiens (data not shown). In multivariable Cox regression analysis, older age, a high WBC and presence of a *FLT3-ITD* were associated with an adverse prognosis, whereas the type of *NPM1* mutation (A versus B versus D versus other) was not significantly associated with OS or RFS (**Table S4 in File S1**). Similar results were obtained for patients not receiving allogeneic transplantation (**text in File S1**).

## Conclusions and Discussion

The aim of this study was to assess the relevance of different *NPM1* mutation types with regard to clinical and molecular patient characteristics and outcome.

In the literature only few published studies have addressed this topic revealing controversial results. Koh et al who investigated only patients without an additional *FLT3-ITD*, found a negative prognostic impact of rare type mutations on OS and duration of CR [4]. Their analyses were based on 18 *NPM1*-mutated de novo AML patients, 13 of which had a type A mutation versus 5 with a rare type mutation. Thus, conclusions are limited due to small sample size and, furthermore, patients were not treated homogeneously within a clinical trial.

In contrast, another study recently presented at the ASH meeting involving 603 *NPM1*-mutated patients with intermediate risk karyotype showed a trend towards a longer OS in rare type versus type A *NPM1* mutations [5]. In the presence of an *FLT3-ITD*, type A patients had a worse OS compared to rare type mutations, whereas there was no difference between these groups in the absence of a *FLT3-ITD*.

Our study of a homogenous cohort with respect to treatment (all patients are treated within one clinical trial) and cytogenetics (all patients had a normal karyotype) did not reveal any difference in molecular and clinical parameters including CR rates, OS and RFS between type A and rare type *NPM1* mutations. Even when our patients were stratified according to *FLT3-ITD* status the type of *NPM1* mutation was not relevant for clinical outcome. This was also true comparing *NPM1* mutation types A, B, D versus other. Moreover, the type of *NPM1* mutation was not an independent prognostic factor in a multivariable analysis.

Except for the addition of a NES causing an aberrant cytoplasmatic localization of the mutated *NPM1* protein, other biochemical consequences of different *NPM1* mutation types on a protein level which might alter the binding of *NPM1* targets have not been fully explored and warrant further investigation.

In light of the low frequency and diversity of rare *NPM1* mutations, however, it is very challenging to assess their individual clinical relevance.

We conclude that the type of *NPM1* mutation does not seem to play a role in risk stratification of cytogenetically normal AML.

## Supporting Information

File S1 **Data supplement.** Text, *NPM1* mutation type does not influence outcome - Subgroup analyses. A) *NPM1* mutation type A versus rare type mutationsin 292 patients receiving chemotherapy only in 292 patients receiving chemotherapy only; in 330 patients with de novo AML; in patients <60 and ≥60 years of age. B) *NPM1* mutation types A versus B versus D versus other mutations in 292 patients receiving chemotherapy only. Table S1, Characteristics of *NPM1*-mutated patients and comparison between those with type A and rare type with or without a *FLT3-ITD*. Table S2, Frequency of different types of *NPM1* mutations. Table S3, Univariable Cox regression for overall survival (OS) and relapse-free survival (RFS). Table S4, Multivariable Cox regression models for OS and RFS in 349 *NPM1*-mutated patients. Figure S1, Overview of patient Selection. Figure S2, Influence of a *FLT3-ITD* in 349 *NPM1*-mutated patients on outcome. (A) OS in 349 *NPM1*-mutated patients. (B) RFS in *NPM1*-mutated patients in CR. Figure S3, Overall Survival (OS) and Relapse-Free Survival (RFS) in 292 patients with cytogenetically normal acute myeloid leukemia and *NPM1* mutation treated in the AMLCG99 study, not receiving allogeneic transplantation. (A) OS in patients with *NPM1* type A mutation versus *NPM1* rare type mutation. (B) OS in patients with *NPM1* type A mutation versus *NPM1* rare type mutation with or without an additional *FLT3-ITD*. (C) RFS in patients with *NPM1* type A mutation versus *NPM1* rare type mutation. (D) RFS in patients with *NPM1* type A mutation versus *NPM1* rare type mutation with or without an additional *FLT3-ITD*. Figure S4, Overall Survival (OS) and Relapse-Free Survival (RFS) in 330 patients with de novo cytogenetically normal acute myeloid leukemia and *NPM1* mutation treated in the AMLCG99 study. (A) OS in patients with *NPM1* type A mutation versus *NPM1* rare type mutation. (B) OS in patients with *NPM1* type A mutation versus *NPM1* rare type mutation with or without an additional *FLT3-ITD*. (C) RFS in patients with *NPM1* type A mutation versus *NPM1* rare type mutation. (D) RFS in patients with *NPM1* type A mutation versus *NPM1* rare type mutation with or without an additional *FLT3-ITD*. Figure S5, Overall Survival (OS) and Relapse-Free Survival (RFS) in 197 patients <60 years and 152 patients ≥60 years with cytogenetically normal acute myeloid leukemia and *NPM1* mutation treated in the AMLCG99 study. (A) OS in patients <60 years with *NPM1* type A mutation versus *NPM1* rare type mutation. (B) OS in patients ≥60 years with *NPM1* type A mutation versus *NPM1* rare type mutation. (C) RFS in patients <60 with *NPM1* type A mutation versus *NPM1* rare type mutation. (D) RFS in patients ≥60 with *NPM1* type A mutation versus *NPM1* rare type mutation.
(PDF)

## References

[1] Falini B, Mecucci C, Tiacci E, Alcalay M, Rosati R, et al (2005) Cytoplasmic nucleophosmin in acute myelogenous leukemia with a normal karyotype. N Engl J Med 352: 254–266 10.1056/NEJMoa041974 15659725

[2] Federici L, Falini B (2013) Nucleophosmin mutations in acute myeloid leukemia: a tale of protein unfolding and mislocalization. Protein Sci 22: 545–556 10.1002/pro.2240 23436734 PMC3649256

[3] Falini B, Bolli N, Shan J, Martelli MP, Liso A, et al (2006) Both carboxy-terminus NES motif and mutated tryptophan(s) are crucial for aberrant nuclear export of nucleophosmin leukemic mutants in NPMc+ AML. Blood 107: 4514–4523 10.1182/blood-2005-11-4745 16455950

[4] Koh Y, Park J, Bae EK, Ahn KS, Kim I, et al (2009) Non-A type nucleophosmin 1 gene mutation predicts poor clinical outcome in de novo adult acute myeloid leukemia: differential clinical importance of *NPM1* mutation according to subtype. Int J Hematol 90: 1–5. 19484332 10.1007/s12185-009-0350-1

[5] Alpermann T, Haferlach C, Dicker F, Eder C, Kohlmann A, et al (2013) Evaluation Of Different *NPM1* Mutations In AML Patients According To Clinical, Cytogenetic and Molecular Features and Impact On Outcome. Blood 122: 51–51 Available: http://bloodjournal.hematologylibrary.org/content/122/21/51.abstract.

[6] Büchner T, Berdel WE, Haferlach C, Haferlach T, Schnittger S, et al (2009) Age-related risk profile and chemotherapy dose response in acute myeloid leukemia: a study by the German Acute Myeloid Leukemia Cooperative Group. J Clin Oncol 27: 61–69 10.1200/JCO.2007.15.4245 19047294

[7] Shaffer LG, McGowan-Jordan J, Schmid M (2013) ISCN 2013: An International System for Human Cytogenetic Nomenclature (2013) - International Standing Committee on Human Cytogenetic Nomenclature. Karger publishers.

[8] Thiede C, Steudel C, Mohr B, Schaich M, Schäkel U, et al (2002) Analysis of *FLT3*-activating mutations in 979 patients with acute myelogenous leukemia: association with FAB subtypes and identification of subgroups with poor prognosis. Blood 99: 4326–4335. 12036858 10.1182/blood.v99.12.4326

[9] Schnittger S, Schoch C, Dugas M, Kern W, Staib P, et al (2002) Analysis of *FLT3* length mutations in 1003 patients with acute myeloid leukemia: correlation to cytogenetics, FAB subtype, and prognosis in the AMLCG study and usefulness as a marker for the detection of minimal residual disease. Blood 100: 59–66. 12070009 10.1182/blood.v100.1.59

[10] Schneider F, Hoster E, Unterhalt M, Schneider S, Dufour A, et al (2012) The *FLT3*ITD mRNA level has a high prognostic impact in *NPM1* mutated, but not in *NPM1* unmutated, AML with a normal karyotype. Blood 119: 4383–4386 10.1182/blood-2010-12-327072 22374696

[11] Dufour A, Schneider F, Metzeler KH, Hoster E, Schneider S, et al (2010) Acute myeloid leukemia with biallelic CEBPA gene mutations and normal karyotype represents a distinct genetic entity associated with a favorable clinical outcome. J Clin Oncol 28: 570–577 10.1200/JCO.2008.21.6010 20038735

[12] Benthaus T, Schneider F, Mellert G, Zellmeier E, Schneider S, et al (2008) Rapid and sensitive screening for CEBPA mutations in acute myeloid leukaemia. Br J Haematol 143: 230–239 10.1111/j.1365-2141.2008.07328.x 18752591

[13] Schnittger S, Schoch C, Kern W, Mecucci C, Tschulik C, et al (2005) Nucleophosmin gene mutations are predictors of favorable prognosis in acute myelogenous leukemia with a normal karyotype. Blood 106: 3733–3739 10.1182/blood-2005-06-2248 16076867

[14] Dohner K, Schlenk RF, Habdank M, Scholl C, Rücker FG, et al (2005) Mutant nucleophosmin (*NPM1*) predicts favorable prognosis in younger adults with acute myeloid leukemia and normal cytogenetics: interaction with other gene mutations. Blood 106: 3740–3746 10.1182/blood-2005-05-2164 16051734

[15] Schemper M, Smith TL (1996) A note on quantifying follow-up in studies of failure time. Control Clin Trials 17: 343–346. 8889347 10.1016/0197-2456(96)00075-x

