# Supplementary material for: The NPM1 Mutation Type Has No Impact on Survival in Cytogenetically Normal AML
Source: PLoS One. 2014 Oct 9;9(10):e109759. doi: 10.1371/journal.pone.0109759 (PMC4192029; doi:10.1371/journal.pone.0109759)
Supplement: File S1 — Data supplement. Text, NPM1 mutation type does not influence outcome - Subgroup analyses. A) NPM1 mutation type A versus rare type mutationsin 292 patients receiving chemotherapy only in 292 patients receiving chemotherapy only; in 330 patients with de novo AML; in patients <60 and ≥60 years of age. B) NPM1 mutation types A versus B versus D versus other mutations in 292 patients receiving chemotherapy only. Table S1, Characteristics of NPM1-mutated patients and comparison between those with type A and rare type with or without a FLT3-ITD. Table S2, Frequency of different types of NPM1 mutations. Table S3, Univariable Cox regression for overall survival (OS) and relapse-free survival (RFS). Table S4, Multivariable Cox regression models for OS and RFS in 349 NPM1-mutated patients. Figure S1, Overview of patient Selection. Figure S2, Influence of a FLT3-ITD in 349 NPM1-mutated patients on outcome. (A) OS in 349 NPM1-mutated patients. (B) RFS in NPM1-mutated patients in CR. Figure S3, Overall Survival (OS) and Relapse-Free Survival (RFS) in 292 patients with cytogenetically normal acute myeloid leukemia and NPM1 mutation treated in the AMLCG99 study, not receiving allogeneic transplantation. (A) OS in patients with NPM1 type A mutation versus NPM1 rare type mutation. (B) OS in patients with NPM1 type A mutation versus NPM1 rare type mutation with or without an additional FLT3-ITD. (C) RFS in patients with NPM1 type A mutation versus NPM1 rare type mutation. (D) RFS in patients with NPM1 type A mutation versus NPM1 rare type mutation with or without an additional FLT3-ITD. Figure S4, Overall Survival (OS) and Relapse-Free Survival (RFS) in 330 patients with de novo cytogenetically normal acute myeloid leukemia and NPM1 mutation treated in the AMLCG99 study. (A) OS in patients with NPM1 type A mutation versus NPM1 rare type mutation. (B) OS in patients with NPM1 type A mutation versus NPM1 rare type mutation with or without an additional FLT3-ITD. (C) RFS in patients wit [file pone.0109759.s001.pdf]

Data Supplement

The *NPM1* mutation type has no impact on survival in cytogenetically normal AML

NPM1 mutation type does not influence outcome - Subgroup analyses
A) NPM1 mutation type A versus rare type mutations

in 292 patients receiving chemotherapy only

In 292 patients receiving chemotherapy only as consolidation treatment, the prognostic effect of a *NPM1-A* versus *NPM1-RA* mutation was not significantly different with respect to OS (p=0.579) and RFS (p=0.630) in all patients **(Figure S3A and S3C)**, nor in patients with a *FLT3*-ITD (OS: p=0.795; RFS: p=0.286) or without a *FLT3*-ITD (OS: p=0.273; RFS: p=0.463) **(Figure S3B and S3D)**. Multivariable Cox regression analyses in these patients revealed the same risk factors as in censored analyses - a high WBC, older age and presence of a *FLT3*-ITD - for a shorter OS and RFS, but the type of *NPM1* mutation (type A versus rare type; type A versus type B versus type D versus other *NPM1* mutation) was not significant.

in 330 patients with de novo AML

Analyses performed in 330 patients with de novo AML revealed a lack of significance of the *NPM1-A* versus *NPM1-RA* mutation type on OS and RFS in all patients **(Figure S4A and S4C)**, those with and those without an additional *FLT3*-ITD **(Figure S4B and S4D)** and in multivariable Cox regression in which only WBC, age and *FLT3*-ITD showed an impact in OS and RFS.

in patients <60 and ≥60 years of age

In 197 patients <60 years and 152 patients ≥60 years, OS (p=0,756, p=0.375, respectively) and RFS (p=0.593, p=0.402, respectively) were not different between patients with *NPM1-A* and *NPM1-RA* **(Figures S5A - S5D)** and the *NPM1* mutation type did not impact on OS and RFS in multivariable analyses.

Similar results were obtained for younger and older patients in *NPM1-A/FLT3*-ITD-, *NPM1-A/FLT3*-ITD+, *NPM1-RA/FLT3*-ITD-, *NPM1-RA/FLT3*-ITD+ subgroups for OS and RFS (data not shown).

B) NPM1 mutation types A versus B versus D versus other mutations in 292 patients receiving chemotherapy only

The *NPM1* mutation types *NPM1-A* versus *NPM1-B* versus *NPM1-D* versus *NPM1-others* did not reveal significant differences with regard to OS and RFS, neither in all patients (p=0.936 p=0.970, respectively), nor in patients with or without an additional *FLT3*-ITD (data not shown). Multivariable Cox regression revealed the same risk factors for an adverse OS and RFS as for all patients.

Table S1: Characteristics of *NPM1*-mutated patients and comparison between those with type A and rare type with or without a *FLT3*-ITD

Characteristic	all (n=349)	*NPM1-A* (n=268)	*NPM1-RA* (n=81)	*p1*	*NPM1-A* / *FLT3*-ITD- (n=161)	*NPM1-A* / *FLT3*-ITD+ (n=107)	*NPM1-RA* / *FLT3*-ITD- (n=47)	*NPM1-RA* / *FLT3*-ITD+ (n=34)	*p2*	*p3*	*p4*
Age, years				0.870					0.799	0.642	0.698
median	57	57	57		58	56	57	58			
range	19-85	19-85	19-78		22-80	19-85	30-75	19-78			
White blood count, G/L (n=347)				0.552					0.171	0.005	<0.001
median	35.7	33.9	41.5		24.1	46.1	18.1	75.0			
range	0.1-798.2	0.1-798.2	0.5-486.0		0.7-798.2	0.1-440.3	0.5-169.0	4.3-486.0			
Platelets, G/L (n=347)				0.714					0.683	0.975	0.440
median	61	63	60		66	52	65	60			
range	5-367	5-367	12-282		5-339	7-367	14-268	12-282			
Hemoglobin level, g/L (n=346)				0.845					0.770	0.951	0.826
median	92	91	92		91	92	90	9.5			
range	42-164	47-164	42-142		53-164	47-139	54-123	42-142			
LDH level, U/l (n=345)				0.302					0.738	0.058	<0.001
median	494	490	557		429	711	434	843			
range	23-14332	23-14332	190-7434		23-5560	171-14332	190-3221	291-7434			
Bone marrow blasts, % (n=327)				0.830					0.589	0.441	0.001
median	85	85	90		84	90	80	90			
range	20-100	20-100	20-100		20-100	20-100	20-100	25-100			
Female sex, n (%)	212 (61)	164 (61)	48 (59)	0.755	92 (57)	72 (67)	26 (55)	22 (65)	0.824	0.781	0.308
Performance status (ECOG), n (%)				0.475					0.139	0.825	0.121
(n=340)	80 (24)	64 (25)	16 (20)		40 (26)	24 (23)	8 (17)	8 (13)			
0	147 (43)	107 (41)	40 (50)		68 (44)	39 (38)	30 (64)	10 (30)			
1	80 (24)	61 (24)	19 (24)		34 (22)	27 (26)	8 (17)	11 (33)			
2	25 (7)	22 (9)	3 (4)		12 (8)	10 (10)	1 (2)	2 (6)			
3	8 (2)	6 (2)	2 (3)		2 (1)	4 (4)	0 (0)	2 (6)			
4											
Origin of AML, n (%)				0.817					0.974	0.678	0.758
de novo	330 (95)	253 (94)	77 (95)		150 (93)	103 (96)	44 (94)	33 (97)			
sAML	12 (3)	10 (4)	2 (3)		8 (5)	2 (2)	2 (4)	0 (0)			
tAML	7 (2)	5 (2)	2 (3)		3 (2)	2 (2)	1 (2)	1 (3)			
FLT3-ITD mRNA mutation level				0.688					NA	0.688	0.688
in patients with *FLT3*-ITD+ (n=124)											
... median	0.42	0.42	0.46		NA	0.42	NA	0.46			
....range	(0.01-0.98)	(0.04-0.97)	(0.01-0.98)			0.04-0.97		0.01-0.98			
mo*CEBPA*+, n (%) (n=348)	12 (3)	11 (4)	1 (1)	0.219	4 (3)	7 (7)	1 (2)	0 (0)	0.904	0.126	0.174
bi*CEBPA*+, n (%) (n=348)	0 (0)	0 (0)	0 (0)	NA	0 (0)	0 (0)	0 (0)	0 (0)	NA	NA	NA

Characteristic	all (n=349)	*NPM1-A* (n=268)	*NPM1-RA* (n=81)	*p*1	*NPM1-A* / *FLT3*-ITD- (n=161)	*NPM1-A* / *FLT3*-ITD+ (n=107)	*NPM1-RA* / *FLT3*-ITD- (n=47)	*NPM1-RA* / *FLT3*-ITD+ (n=34)	*p*2	*p*3	*p*4
Induction regimen, n (%)				0.941					0.901	0.728	0.743
TAD	58 (17)	44 (16)	15 (17)		29 (18)	15 (14)	10 (21)	4 (12)			
HAM	69 (20)	54 (20)	15 (19)		34 (21)	20 (19)	8 (17)	7 (21)			
TAD-HAM	121 (35)	91 (34)	30 (37)		50 (31)	41 (38)	14 (30)	16 (47)			
HAM-HAM	101 (29)	79 (30)	22 (27)		48 (30)	31 (29)	15 (32)	7 (21)			
Allogeneic transplantation	57 (16)	45 (17)	12 (15)	0.673	25 (16)	20 (19)	7 (15)	5 (15)	0.916	0.596	0.885
OS, months				0.589					0.262	0.646	<0.001
median	45.6	40.7	73.3		79.7	10.1	97.8	10.7			
events, n (%)	151 (57)	153 (57)	45 (56)		58 (36)	57 (53)	15 (32)	21 (62)			
CR	260 (75)	201 (75)	59 (73)	0.696	121 (75)	80 (75)	38 (81)	21 (62)	0.418	0.143	0.268
RFS, years (n=258)				0.827					0.418	0.256	<0.001
median	37.1	35.7	44.0		53.9	7.0	81.4	7.0			
events, n (%)	115 (55)	86 (43)	29 (49)		44 (37)	42 (53)	15 (39)	14 (67)			

Abbreviations: bi*CEBPA+*, biallelic mutation of the CCAAT/enhancer-binding protein alpha gene; CR, complete remission; de novo AML; origin of AML de novo; ECOG, performance status according to the Eastern Cooperative Oncology Group; ELN, European Leukemia Net; *FLT3*-ITD+, presence of an internal tandem duplication in the fms-related tyrosine 3 gene; *FLT3*-ITD-, absence of an internal tandem duplication in the fms-related tyrosine 3 gene; HAM, high-dose cytarabine, mitoxantrone; LDH, lactase dehydrogenase; mo*CEBPA+*, mutation of the CCAAT/enhancer-binding protein alpha gene; n, number; NA, not applicable; *NPM1+,* presence of a mutation in the nucleophosmin gene; *NPM1-A*, mutation in the nucleophosmin gene leading to the insertion of the tetranucleotide TCTG; *NPM1-RA,* mutation in the nucleophosmin gene other than type A, OS, Overall survival; RFS, Relapse-free survival; sAML, secondary AML; TAD, thioguanine, cytarabine, daunorubicin; tAML, therapy-related AML, *v* ,versus.

*p*1: *NPM1-A* versus *NPM1-RA*
*p*2: *NPM1-A* / *FLT3*-ITD- versus *NPM1-RA* / *FLT3*-ITD-
*p*3: comparison: *NPM1-A* / *FLT3*-ITD+ versus *NPM1-RA* / *FLT3*-ITD+
*p*4: comparison: all four *NPM1*-type/ *FLT3*-ITD combinations

Table S2: Frequency of different types of *NPM1* mutations

type<br>nucleotide	number	frequency (%)	Wt<br>253-260	Insert	Wt<br>261-265	Insert	Wt
A	268	76.8	gatctctg	tctg	gcagt	-	ggaggaagtctcttt
B	19	5.4	gatctctg	catg	gcagt	-	ggaggaagtctcttt
D	31	8.9	gatctctg	cctg	gcagt	-	ggaggaagtctcttt
G·	2	0.6	gatctctg	cagg	gcagt	-	ggaggaagtctcttt
J†	4	1.1	gatctctg	tatg	gcagt	-	ggaggaagtctcttt
K_m	3	0.9	gatctctg	ccgg	gcagt	-	ggaggaagtctcttt
M_m	1	0.3	gatctctg	-	gcag	agga	tggaggaagtctcttt
N_m	4	1.1	gatctctg	ccag	gcagt	-	ggaggaagtctcttt
P_m	6	1.7	gatctctg	cttg	gcagt	-	ggaggaagtctcttt
Q_m	1	0.3	gatctctg	tcgg	gcagt	-	ggaggaagtctcttt
13	1	0.3	gatctctg	taag	gcagt	-	ggaggaagtctcttt
M*	1	0.3	gatctctg	ccgcct	agt	-	ggaggaagtctcttt
P*	2	0.6	gatctctg	caag	gcagt	-	ggaggaagtctcttt
S*	1	0.3	gatctct	ct	gcag	cct	tggaggaagtctcttt
T*	1	0.3	gatctctg	ccgc	gcagt	-	ggaggaagtctcttt
U*	1	0.3	gatctctg	-	gcagtg	tttttccc	aagtctcttt
W*	1	0.3	gatctctg	caca	gcagt	-	ggaggaagtctcttt
X*	1	0.3	gatct	ttgc	ctggcagt	-	ggaggaagtctcttt
Y*	1	0.3	gatctctg	tagg	gcagt	-	ggaggaagtctcttt

Mutations A to D refer to mutations identified by Falini et al.[1]

Mutation G· refers to the mutation identified by Cazzaniga et al.[2]

Mutation J†· refers to the mutation identified by Suzuki et al.[3]

Mutations K_m to Q_m refer to mutations identified by Schnittger et al.[4]

Mutation 13 refers to the mutations identified by Döhner et al.[5]

Mutations M* to Y* refer to newly identified mutations identified by our group.

Abbreviations: Wt, wild-type

Table S3: Univariable Cox regression for overall survival (OS) and relapse-free survival (RFS)

		OS				RFS			
Variable	Comparison	n	Hazard Ratio	95% CI	p	n	Hazard Ratio	95% CI	p
Age (years)	+10 years	349	1.5	1.3 -1.8	<0.001	259	1.2	1.1-1.4	<0.001
WBC (10^9/l)	10 fold	349	2.0	1.5-2.7	<0.001	259	2.0	1.4-2.8	<0.001
Platelets (10^9/l)	10 fold	349	0.7	0.447-1.023	0.06	259	0.6	0.412-1.012	0.06
Hb (g/l)	+1 g/L	349	1.0	0.994-1.011	0.55	259	1.0	0.990-1.009	0.92
LDH level (U/l)	10 fold	349	2.6	1.7-4.1	<0.001	259	3.1	1.8-5.5	<0.001
BM blasts (%)	+1%	349	1.0	0.998-1.019	0.10	259	1.0	0.998-1.022	0.11
Sex	male vs. female	349	0.8	0.704-0.985	0.033	259	0.9	0.8-1.1	0.39
Performance status (ECOG)	2-4 vs. 0,1	349	2.0	1.4-2.7	<0.001	259	1.6	1.1-2.4	0.014
Origin of AML	de novo vs. non de novo	349	0.9	0.4-1.7	0.70	259	1.2	0.6-3.1	0.63
NPM1 mutation type	*NPM1-A* vs. *NPM1-RA*	349	1.1	0.8-1.6	0.59	259	1.0	0.7-1.6	0.83
NPM1 mutation type	*NPM1-A* vs. *NPM1-B* vs. *NPM1-D* vs. *NPM1-others*	349	0.9	0.7-1.2	0.62	259	1.0	0.8-1.3	0.89
FLT3-ITD	pos. vs. neg	349	2.2	1.6-3.0	<0.001	259	2.7	1.8-3.9	<0.001
interaction term *NPM1*/*FLT3*-ITD	pos./pos. vs. pos./neg.	349	2.2	1.6-3.1	<0.001	259	2.8	2.0-4.1	<0.001
CEBPA	mo*CEBPA* vs. wt	349	0.5	0.2-1.6	0.27	259	0.7	0.2-2.1	0.47

Abbreviations: BM, bone marrow; *CEBPA*, CCAAT/enhancer-binding protein alpha gene; CI, confidence interval; ECOG, Eastern Cooperative Oncology Group; *FLT3*-ITD, internal tandem duplication of the *FLT3* gene; Hb, hemoglobin level; HR, hazard ratio; negative, absence of *FLT3*-ITD; LDH, lactase dehydrogenase level; mo*CEBPA*, monoallelic *CEBPA* mutation; *NPM1*, nucleophosmin gene; *NPM1-A*, mutation in the nucleophosmin gene leading to the insertion of the tetranucleotide TCTG; *NPM1-B*, mutation in the nucleophosmin gene consisting of an insertion of the tetranucleotide CATG, *NPM1-D*, mutation in the nucleophosmin gene consisting of an insertion of the tetranucleotide CCTG, *NPM1-other*, mutation in the nucleophosmin gene other than *NPM1* mutation types A, B, D. *NPM1-RA*, mutation in the nucleophosmin gene other than type A; OS, Overall survival; p, p value; positive, presence of *FLT3*-ITD; RFS, Relapse-free survival; WBC, white blood cell count, wt, wildtype.

Table S4: Multivariable Cox regression models for OS and RFS in 349 *NPM1*-mutated patients

independent prognostic factors	comparison	OS			RFS		
		HR	95% CI	p	HR	95% CI	p
Age	per 10 years	1.71	1.44-2.03	<0.001	1.55	1.28-1.87	<0.001
WBC	10^9/ L, per 10-fold increase	1.63	1.16-2.30	0.005	1.71	1.15-2.56	0.009
FLT3-ITD	positive versus negative	2.04	1.40-2.96	<0.001	2.45	1.60-3.75	<0.001
NPM1 mutation type	*NPM1-A* vs *NPM1-B* vs *NPM1-D* vs *NPM1-other*			0.879			0.854
	NPM1-A vs NPM1-other	1.18	0.63-2.19	0.611	0.88	0.45-1.76	0.727
	NPM1-A vs *NPM1-B*	0.83	0.40-1.72	0.618	0.72	0.32-1.58	0.409
	NPM1-A vs NPM1-D	1.13	0.61-2.09	0.691	0.98	0.49-1.95	0.961

White blood cell count, platelet count, hemoglobin level, lactase dehydrogenase level, bone marrow blasts, de novo AML versus non de novo AML, performance status, sex, age, type A versus rare type *NPM1* mutation, *FLT3*-ITD, monoallelic *CEBPA* mutations, biallelic *CEBPA* mutations were included in the Cox regression models for OS and RFS with backward elimination. The analyses were performed using 313 patients for OS and 227 RFS who had data for all these variables. A p-value of <0.05 was considered as indicating significant differences. All parameters that did not have a significant impact on OS or RFS are not shown in the table, except for the *NPM1* mutation type.

Abbreviations: *CEBPA*, CCAAT/enhancer-binding protein alpha gene; CI, confidence interval; *FLT3*-ITD, internal tandem duplication of the *FLT3* gene; HR, hazard ratio; negative, absence of *FLT3*-ITD; *NPM1-A*, mutation in the nucleophosmin gene consisting of an insertion of the tetranucleotide TCTG; *NPM1-B*, mutation in the nucleophosmin gene consisting of an insertion of the tetranucleotide CATG, *NPM1-D*, mutation in the nucleophosmin gene consisting of an insertion of the tetranucleotide CCTG, *NPM1-other*, mutation in the nucleophosmin gene other than *NPM1* mutation types A, B, D; OS, Overall survival; p, p value; positive, presence of *FLT3*-ITD; RFS, Relapse-free survival; vs, versus; WBC, white blood cell count.

Figure S1: Overview of patient Selection

Abbreviations: AML, acute myeloid leukemia; AMLCG, AML Cooperative Group; MDS, myelodysplastic syndrome; *NPM1*, mutation in the nucleophosmin gene.

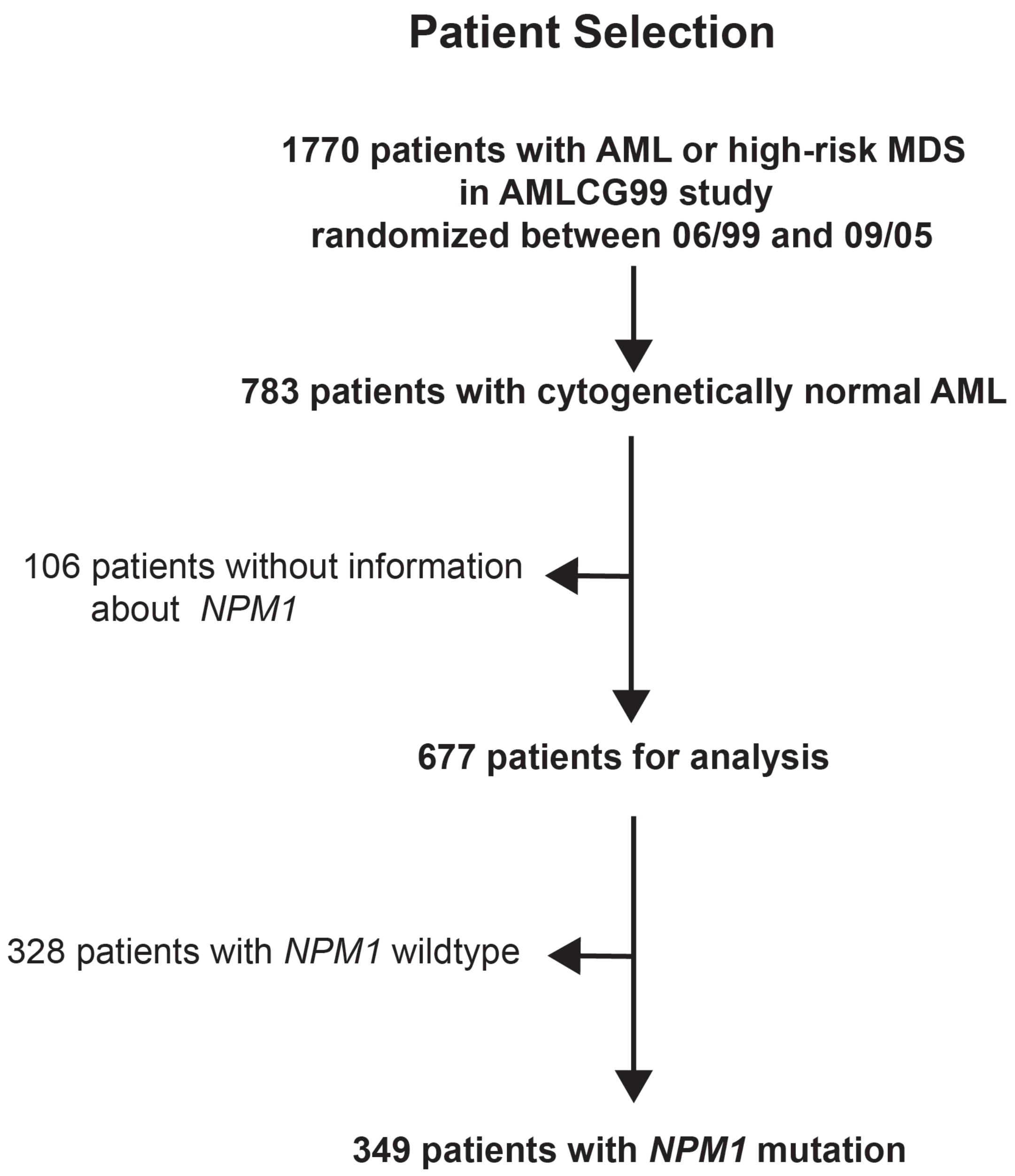

Figure S2: Influence of a *FLT3*-ITD in 349 *NPM1*-mutated patients on outcome. (A) OS in 349 *NPM1*-mutated patients. (B) RFS in *NPM1*-mutated patients in CR.

Abbreviations: CR, complete remission; *FLT3*-ITD+, presence of an internal tandem duplication in the fms-related tyrosine 3 gene; *FLT3*-ITD-, absence of an internal tandem duplication in the fms-related tyrosine 3 gene; *NPM1*, mutation in the nucleophosmin gene

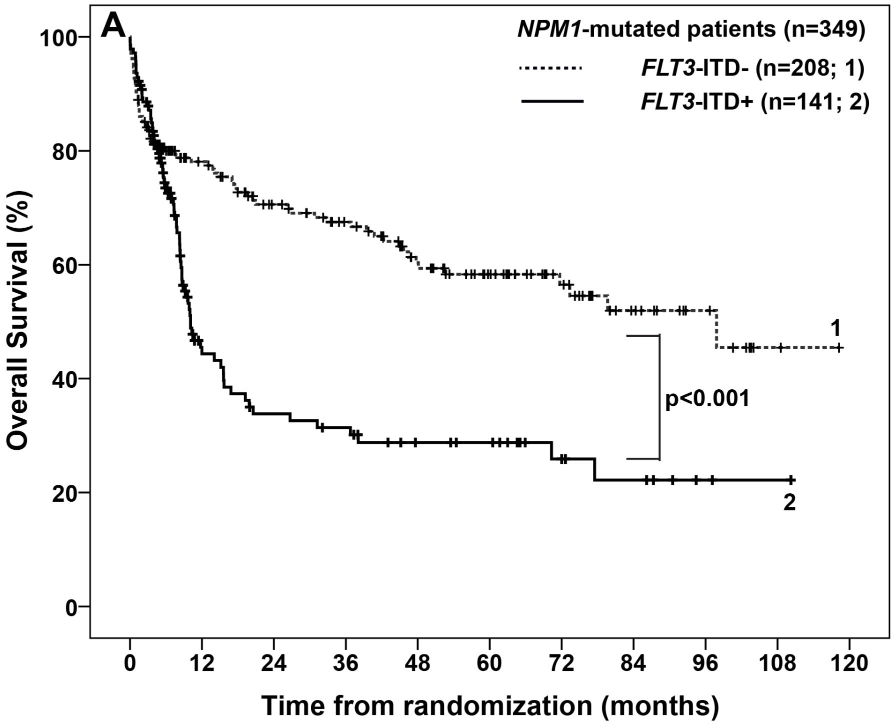

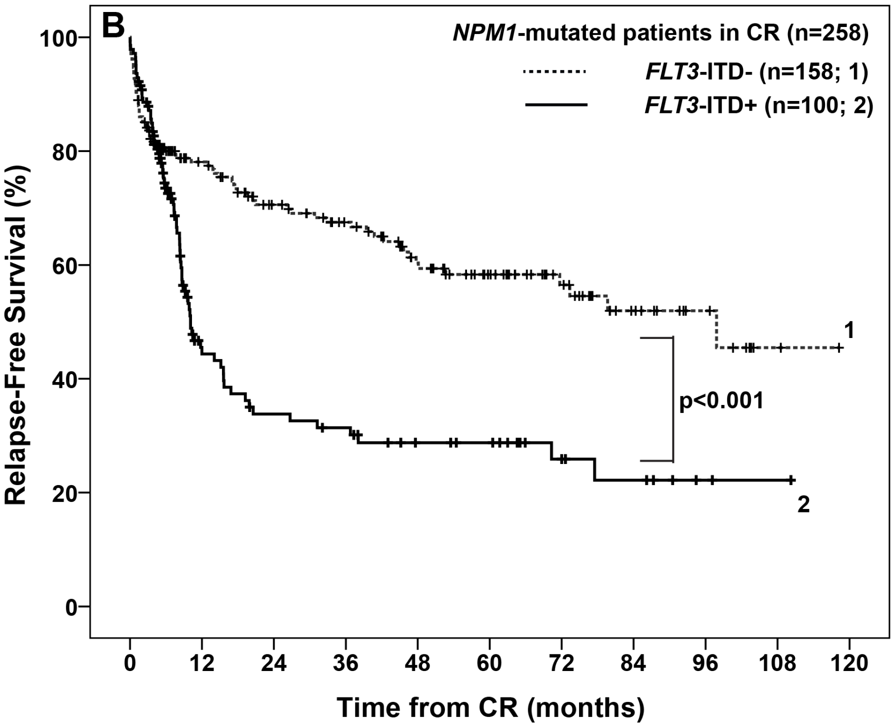

Figure S3: Overall Survival (OS) and Relapse-Free Survival (RFS) in 292 patients with cytogenetically normal acute myeloid leukemia and *NPM1* mutation treated in the AMLCG99 study, not receiving allogeneic transplantation. (A) OS in patients with *NPM1* type A mutation versus *NPM1* rare type mutation. (B) OS in patients with *NPM1* type A mutation versus *NPM1* rare type mutation with or without an additional *FLT3*-ITD. (C) RFS in patients with *NPM1* type A mutation versus *NPM1* rare type mutation. (D) RFS in patients with *NPM1* type A mutation versus *NPM1* rare type mutation with or without an additional *FLT3*-ITD.

Abbreviations: CR, complete remission; *FLT3*-ITD+, presence of an internal tandem duplication in the fms-related tyrosine 3 gene; *FLT3*-ITD-, absence of an internal tandem duplication in the fms-related tyrosine 3 gene; *NPM1-A*, mutation in the nucleophosmin gene consisting of an insertion of the tetranucleotide TCTG; *NPM1-RA*, mutation in the nucleophosmin gene other than type A.

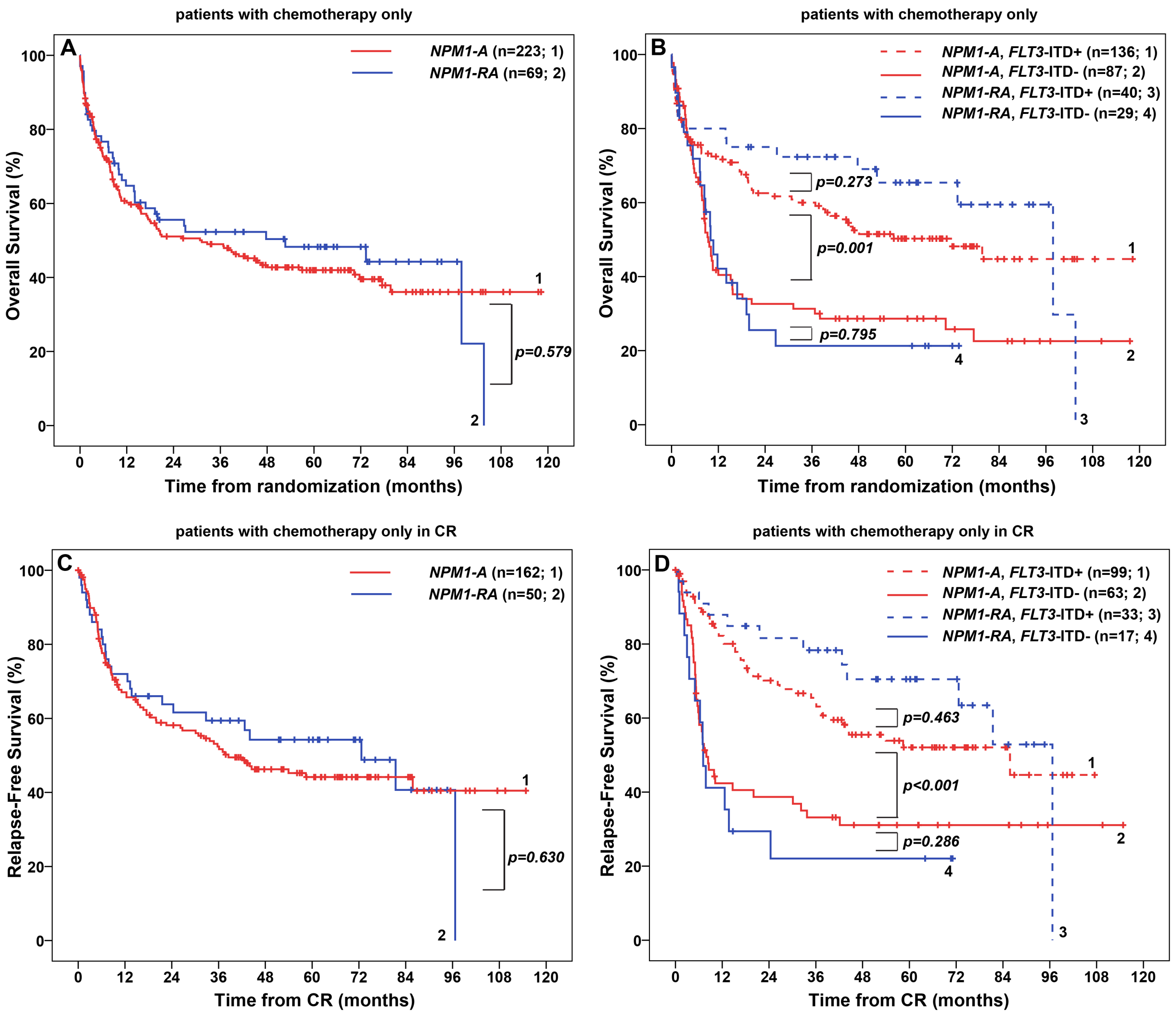

Figure S4: Overall Survival (OS) and Relapse-Free Survival (RFS) in 330 patients with de novo cytogenetically normal acute myeloid leukemia and *NPM1* mutation treated in the AMLCG99 study. (A) OS in patients with *NPM1* type A mutation versus *NPM1* rare type mutation. (B) OS in patients with *NPM1* type A mutation versus *NPM1* rare type mutation with or without an additional *FLT3*-ITD. (C) RFS in patients with *NPM1* type A mutation versus *NPM1* rare type mutation. (D) RFS in patients with *NPM1* type A mutation versus *NPM1* rare type mutation with or without an additional *FLT3*-ITD.

Abbreviations: CR, complete remission; *FLT3*-ITD+, presence of an internal tandem duplication in the fms-related tyrosine 3 gene; *FLT3*-ITD-, absence of an internal tandem duplication in the fms-related tyrosine 3 gene; *NPM1-A*, mutation in the nucleophosmin gene consisting of an insertion of the tetranucleotide TCTG; *NPM1-RA*, mutation in the nucleophosmin gene other than type A.

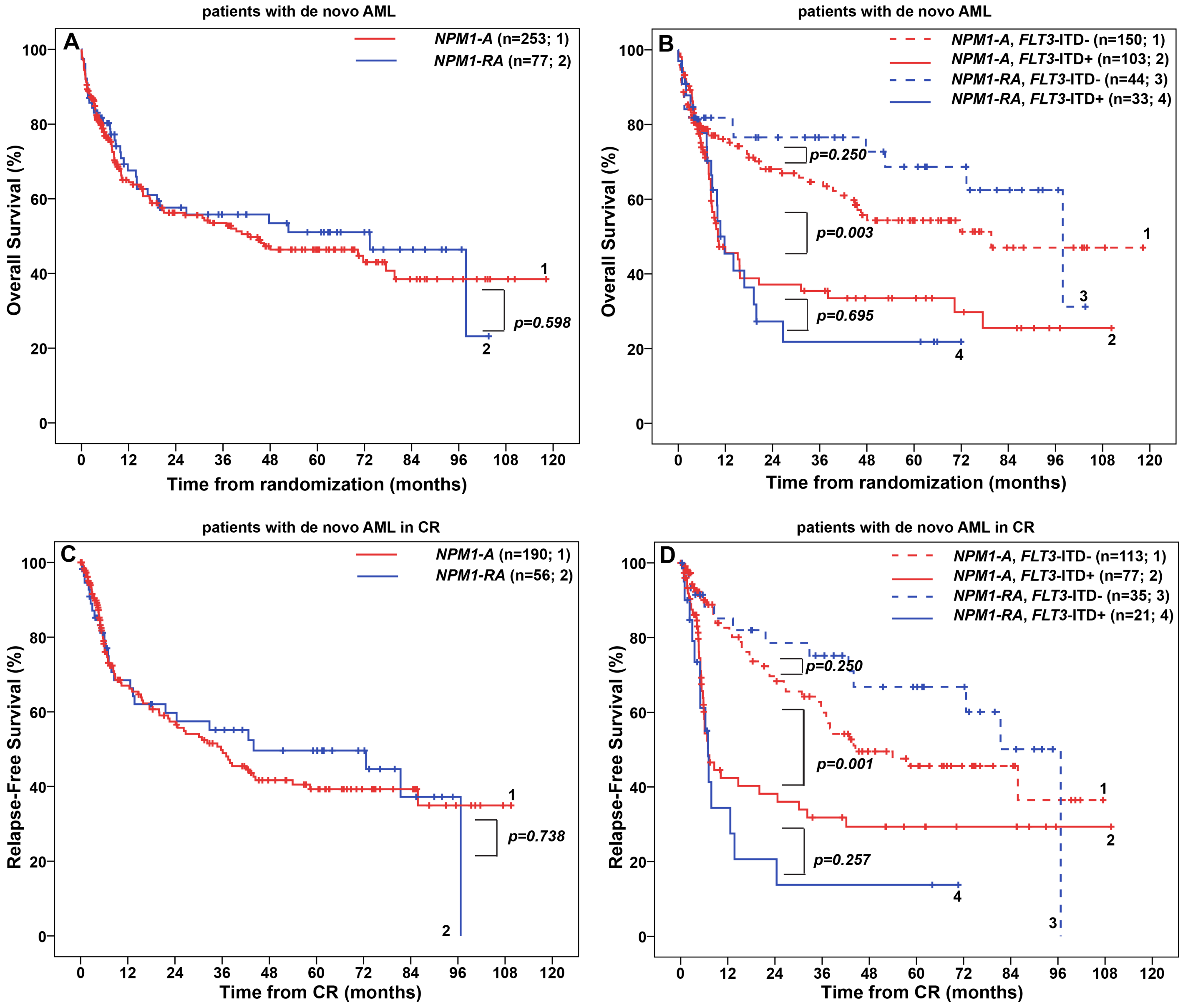

Figure S5: Overall Survival (OS) and Relapse-Free Survival (RFS) in 197 patients <60 years and 152 patients ≥60 years with cytogenetically normal acute myeloid leukemia and *NPM1* mutation treated in the AMLCG99 study. (A) OS in patients <60 years with *NPM1* type A mutation versus *NPM1* rare type mutation. (B) OS in patients ≥60 years with *NPM1* type A mutation versus *NPM1* rare type mutation. (C) RFS in patients <60 with *NPM1* type A mutation versus *NPM1* rare type mutation. (D) RFS in patients ≥60 with *NPM1* type A mutation versus *NPM1* rare type mutation.

Abbreviations: CR, complete remission; *FLT3*-ITD+, presence of an internal tandem duplication in the fms-related tyrosine 3 gene; *FLT3*-ITD-, absence of an internal tandem duplication in the fms-related tyrosine 3 gene; *NPM1-A*, mutation in the nucleophosmin gene consisting of an insertion of the tetranucleotide TCTG; *NPM1-RA*, mutation in the nucleophosmin gene other than type A.

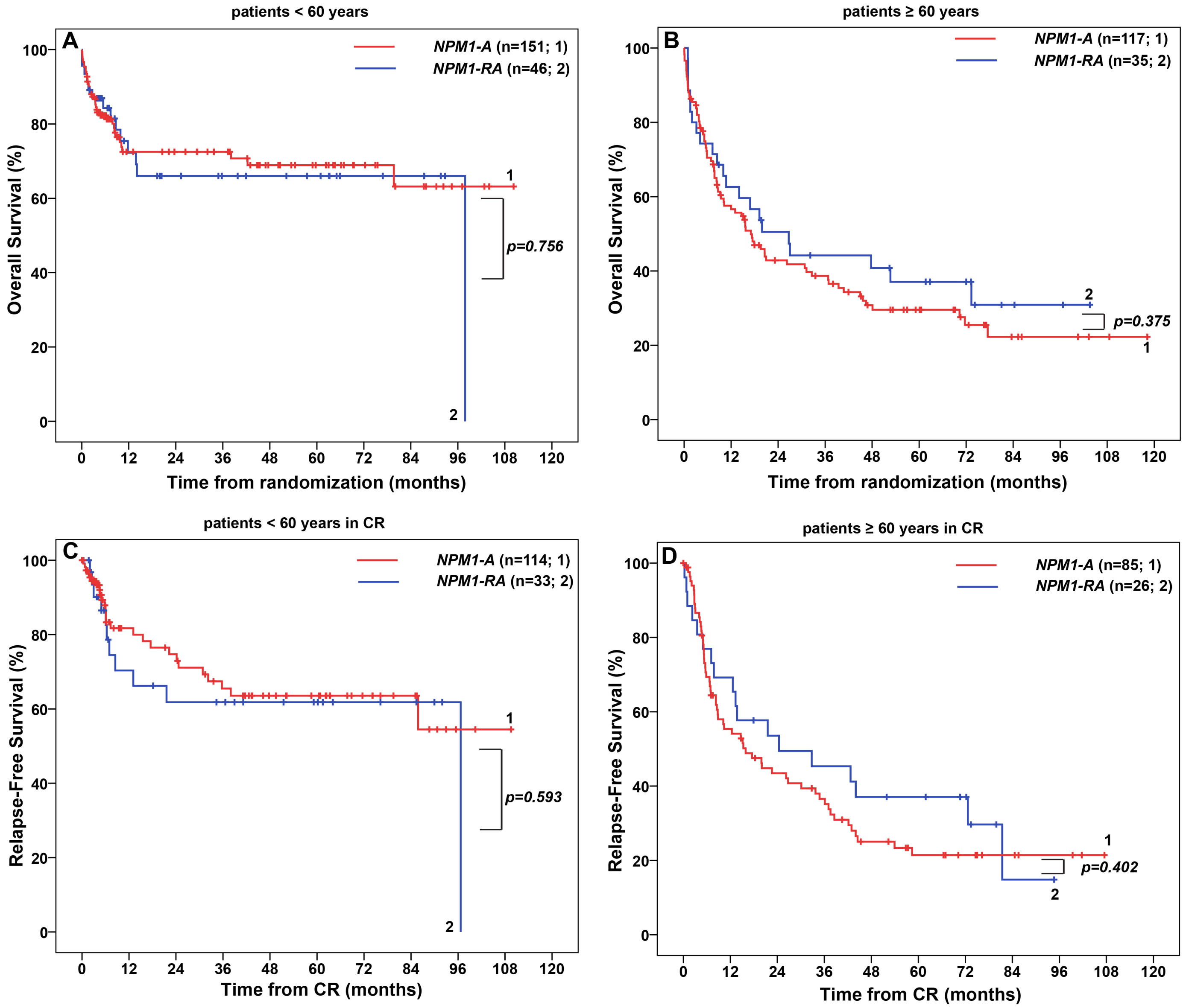

References:

1. Falini B, Mecucci C, Tiacci E, Alcalay M, Rosati R, Pasqualucci L, et al. Cytoplasmic nucleophosmin in acute myelogenous leukemia with a normal karyotype. N Engl J Med. 2005 Jan 20;352(3):254–66.

2. Cazzaniga G, Grazia Dell'Oro M, Mecucci C, Giarin E, Masetti R, Rossi V, et al. Nucleophosmin mutations in childhood acute myelogenous leukemia with normal karyotype. Blood. 2005 Aug 15;106(4):1419–22.

3. Suzuki T, Kiyoi H, Ozeki K, Tomita A, Yamaji S, Suzuki R, et al. Clinical characteristics and prognostic implications of NPM1 mutations in acute myeloid leukemia. Blood. 2005 Oct 15;106(8):2854–61.

4. Schnittger S, Schoch C, Kern W, Mecucci C, Tschulik C, Martelli MF, et al. Nucleophosmin gene mutations are predictors of favorable prognosis in acute myelogenous leukemia with a normal karyotype. Blood. 2005 Dec 1;106(12):3733–9.

5. Dohner K, Schlenk RF, Habdank M, Scholl C, Rücker FG, Corbacioglu A, et al. Mutant nucleophosmin (NPM1) predicts favorable prognosis in younger adults with acute myeloid leukemia and normal cytogenetics: interaction with other gene mutations. Blood. 2005 Dec 1;106(12):3740–6.
